# Mitochondrial DNA content in breast cancer: Impact on *in vitro* and *in vivo* phenotype and patient prognosis

**DOI:** 10.18632/oncotarget.8688

**Published:** 2016-04-11

**Authors:** Marjolein J.A. Weerts, Anieta M. Sieuwerts, Marcel Smid, Maxime P. Look, John A. Foekens, Stefan Sleijfer, John W.M. Martens

**Affiliations:** ^1^ Department of Medical Oncology and Cancer Genomics, Erasmus MC Cancer Institute, Erasmus University Medical Center, Rotterdam, The Netherlands

**Keywords:** breast cancer, mitochondrial DNA, mtDNA content, epithelial-to-mesenchymal transition, distant metastasis

## Abstract

Reduced mitochondrial DNA (mtDNA) content in breast cancer cell lines has been associated with transition towards a mesenchymal phenotype, but its clinical consequences concerning breast cancer dissemination remain unidentified. Here, we aimed to clarify the link between mtDNA content and a mesenchymal phenotype and its relation to prognosis of breast cancer patients. We analyzed mtDNA content in 42 breast cancer cell lines and 207 primary breast tumor specimens using a combination of quantitative PCR and array-based copy number analysis. By associating mtDNA content with expression levels of genes involved in epithelial-to-mesenchymal transition (EMT) and with the intrinsic breast cancer subtypes, we could not identify a relation between low mtDNA content and mesenchymal properties in the breast cancer cell lines or in the primary breast tumors. In addition, we explored the relation between mtDNA content and prognosis in our cohort of primary breast tumor specimens that originated from patients with lymph node-negative disease who did not receive any (neo)adjuvant systemic therapy. When patients were divided based on the tumor quartile levels of mtDNA content, those in the lowest quarter (≤ 350 mtDNA molecules per cell) showed a poorer 10-year distant metastasis-free survival than patients with > 350 mtDNA molecules per cell (HR 0.50 [95% CI 0.29–0.87], *P* = 0.015). The poor prognosis was independent of established clinicopathological markers (HR 0.54 [95% CI 0.30–0.97], *P* = 0.038). We conclude that, despite a lack of evidence between mtDNA content and EMT, low mtDNA content might provide meaningful prognostic value for distant metastasis in breast cancer.

## INTRODUCTION

Mitochondria play a role in many cellular processes including oxidative phosphorylation, redox homeostasis, controlling calcium levels for regulation of signal transduction pathways, and the intrinsic apoptotic pathway [1]. The mitochondria contain their own genome – mtDNA – encoding their own translational machinery and 13 crucial proteins for the oxidative phosphorylation system. Related to energy needs, numerous mtDNA molecules may exist in a single cell. This number is not only dependent on the amount of mitochondria per cell but also on the number of mtDNA molecules per mitochondrion. Broad ranges in mtDNA content have been reported, from a few molecules in embryonic and pluripotent stem cells [2, 3] up to several thousands in subcutaneous adipocytes [4] or cardiac myocytes [5]. The cell-specific mtDNA content is assumed to be fairly stable under physiological conditions but can be altered by stress such as exogenous toxins [6], viral infection [7] and by genetic mutations [8]. The effects of changes in mtDNA content are illustrated in several mtDNA depletion syndromes [9], which are all characterized by impaired energy production.

Several studies examined mtDNA content in the context of cancer but so far no clear picture has emerged. In preclinical models, depletion of mtDNA yielded both increased and decreased *in vitro* tumorigenic phenotypes [10–17]. The *in vivo* findings using mouse xenografts are indecisive as well, as both gain and loss of tumorigenic potential upon mtDNA depletion has been reported [16–21]. Additionally, contradictory findings have been described for mtDNA content in human tumor specimens compared to their healthy counterparts in multiple cancer types (as reviewed in [22, 23]).

With regard to breast cancer, the impact of the mtDNA content on phenotype, prognosis and drug response has been investigated in several studies. Lower mtDNA content is observed in approximately 70% of breast cancer specimens when compared to their surrounding normal epithelium [24–31]. There are indications that low mtDNA content in breast cancer may yield a more aggressive phenotype and altered therapy responses. First, depletion of mtDNA in *in vitro* models affects the mRNA and protein expression levels of several genes involved in epithelial-to-mesenchymal transition (EMT) [12, 14]. The transition towards the mesenchymal phenotype has been implied as an essential mechanism contributing to cancer dissemination [32]. Consequently, low mtDNA content as a marker for the mesenchymal phenotype potentially identifies tumor aggressiveness. Second, a link between reduced mtDNA content and resistance to anti-estrogen regimens has been established in *in vitro* models [33]. Nevertheless, no association between estrogen receptor status and mtDNA content was observed in breast tumors [24–29]. Also, reduced mtDNA content was linked to a shift in drug response for breast cancer cell lines [17, 24, 34]. An *in vitro* reduction in mtDNA content revealed increased sensitivity to cisplatin [17] and doxorubicin [24], but also decreased sensitivity to vincristine, paclitaxel and – in contrast to a previous study – doxorubicin [34]. In a small patient cohort, low mtDNA content was associated with longer disease-free survival in patients receiving adjuvant chemotherapy, whereas this was not the case for patients not receiving adjuvant treatment [24]. Few additional studies reported on breast cancer patient disease free- or overall survival in relation to tumorous mtDNA content [25–27]. However, these studies had either relatively small sample sizes or no information about treatments administered, the mtDNA content determination methods varied, and results were inconclusive.

Here, we further explore the putative link between mtDNA content and prognostic features in breast cancer. In a broad panel of human breast cancer cell lines the link between mtDNA content and a mesenchymal phenotype was studied by correlating it with expression levels of EMT-related genes and with the intrinsic subtypes of breast cancer [35, 36]. In a well-defined patient cohort of primary breast tumor specimens [37], tumor mtDNA content was examined in relation to expression levels of EMT-related genes, to the intrinsic subtypes, as well as to established clinicopathological variables. Primarily, in our cohort of primary breast cancer patients with lymph node-negative disease who did not receive any (neo)adjuvant systemic therapy, we examined the prognostic value of mtDNA content using distant metastasis-free survival as the main endpoint.

## RESULTS

### mtDNA content in breast cancer cell lines and primary tumor specimens

In total, we analyzed DNA extracts from 42 breast cancer cell lines and 207 primary tumor specimens. Multiplex real time quantitative PCR (qPCR) targeting a nuclear-encoded and a mitochondrial-encoded gene combined with array-based copy number changes of the nuclear-encoded gene to correct for sample specific somatic variation at the reference locus was used to obtain the mtDNA content in the DNA extracts of these samples. Inter-assay variability of the multiplex qPCR assay was monitored using the calibration curves taken along in each run (*n* = 7). Amplification in the calibration curve samples was linear between 0.16 and 16 ng DNA per reaction with mean efficiencies and standard error of 97.6 ± 4.4% for nuclear encoded HMBS and 91.5 ± 5.2% for mitochondrial encoded *MT-TL1*. Copy number variation of the nuclear encoded *HMBS* gene was observed in 39% of the breast cancer cell lines including 1 with homozygous loss, 12 with heterozygous loss and 3 with gain, and in 14% of the primary tumor specimens including 20 with heterozygous loss and 10 with gain. Because of a homozygous *HMBS* loss in SUM1315MO2, this cell line was excluded from further analysis. Because of absence of *HMBS* qPCR signal amplification in three primary tumor specimens, these samples were excluded from further analysis as well. The median mtDNA content and interquartile ranges (IQR) in the 41 breast cancer cell lines and in the 204 primary breast tumor specimens were respectively 489 (IQR 360) and 462 (IQR 294) mtDNA molecules per cell.

### mtDNA content and the mesenchymal characteristics

*In vitro* reduction of mtDNA content has been linked to changes in expression of the EMT-related genes *CDH1* [12, 14], *CDH2* [14], *ESRP1* [14], *FN1* [14], *MMP9* [14], *SNAI1* [14], *SNAI2* [14], *TGFB1* [12], *TGFBR1* [12], *TWIST1* [14] and *VIM* [12, 14]. To address whether a more mesenchymal phenotype is a physiological characteristic linked to low mtDNA content [12, 14], we analyzed the relation between mtDNA content and the RNA expression levels of genes related to EMT. Expression data for the above mentioned genes were available for 40 of the 41 breast cancer cell lines and all 204 primary breast tumor specimens. Expression data of *TGFBR1* was excluded because the probe gave expression levels close to background noise. Correlation between gene expression levels and mtDNA content did not exceed a correlation coefficient ρ of 0.35, and we could not demonstrate statistical significance after correction for multiple testing (all *P* > 0.027, Supplementary Table S1) in the breast cancer cell lines. In our primary breast tumor specimens, correlation between mtDNA content and the expression of *ESRP1* (ρ = 0.25, *P* < 0.001), *SNAI1* (ρ = 0.23, *P* < 0.001) and *TGFB1* (ρ = 0.18, *P* < 0.01) was statistically significant after correction for multiple testing (Supplementary Table S1). To further explore the link between mtDNA content and EMT, we analyzed the association between mtDNA content and the intrinsic subtypes of breast cancer, which have been assigned with epithelial or mesenchymal characteristics [36, 38, 39]. Comparisons between the intrinsic subtypes for both the breast cancer cell lines as well as the primary tumor specimens did not show differences in mtDNA content among the subtypes (*P* > 0.05) (Table 1).

**Table 1:** mtDNA content in the intrinsic breast cancer subtypes

	Subtype	*n* (%)	mtDNA content (IQR)	*P*-value
Breast cancer cell lines	Basal	5 (12.5%)	269 (149)	0.1^†^
	ERBB2	7 (17.5%)	620 (521)	
	Luminal	19 (47.5%)	518 (359)	
	Normal	9 (22.5%)	489 (142)	
Primary breast tumor specimens	Basal	65 (31.8%)	454 (287)	0.8^†^
	ERBB2	35 (17.2%)	566 (351)	
	Luminal A	56 (27.5%)	423 (224)	
	Luminal B	40 (19.6%)	514 (343)	
	Normal	8 (3.9%)	377 (286)	

† Median mtDNA content [number of mtDNA molecules per cell] with interquartile range (IQR) for each group and corresponding probabilities (*P* value) for equal distribution using Kruskal-Wallis one-way analysis of variance.

### Association of tumor mtDNA content with established prognostic clinicopathological variables

In our patient cohort, we analyzed tumor mtDNA content in relation to patient age at diagnosis, menopausal status, tumor size, histological grade, estrogen receptor status, progesterone receptor status and *ERBB2* amplification (Table 2). Because the currently used conventional histological grade (modified Bloom-Richardson) was not available for nearly 25% of our cohort – samples originated from multiple hospitals and from time periods when histological grading according to Bloom-Richardson was not common – we included a molecular grading system shown to be equivalent, the qRT-PCR genomic grade index (GGI) [40]. There were no statistically significant associations between the tumor mtDNA content and age at diagnosis, menopausal status, estrogen receptor status, progesterone receptor status or *ERBB2* amplification status (*P* > 0.05). However, tumors smaller than 2 cm had statistically significant lower mtDNA content (median 421 mtDNA molecules per cell) compared to tumors larger than 2 cm (median 514 mtDNA molecules per cell) (Mann-Whitney *P* = 0.019). In addition, tumor mtDNA content varied between the GGI groups (Kruskal-Wallis *P* = 0.028), with the highest mtDNA content in the GGI group representing poorly differentiated high grade 3 tumors (median 523 mtDNA molecules per cell). However, we did not observe a significant trend in tumor mtDNA content across the GGI groups (Cuzick's test for trend *P* = 0.066).

**Table 2:** Association between clinicopathological variables and mtDNA content

Variable	Group	*n* (%)	mtDNA content (IQR)	*P*-value
Age at diagnosis	≤ 40	21 (10.3%)	491 (532)	0.21^†^
	> 40–55	88 (43.1%)	433 (273)	
	> 55–70	64 (31.4%)	466 (259)	
	> 70	31 (15.2%)	546 (490)	
Menopausal status	Pre	99 (48.5%)	427 (323)	0.15^#^
	Post	105 (51.5%)	500 (280)	
Tumor size	≤ 2 cm	99 (48.5%)	421 (280)	0.019^#^
	> 2 cm	105 (51.5%)	514 (382)	
Genomic Grade Index	1	35 (17.2%)	440 (225)	0.028^†^
	2	59 (32.4%)	410 (260)	
	3	103 (50.5%)	523 (389)	
Estrogen receptor status	Negative	87 (42.7%)	483 (328)	0.12^#^
	Positive	115 (56.4%)	424 (290)	
Progesterone receptor status	Negative	97 (47.5%)	480 (335)	0.073^#^
	Positive	96 (47.1%)	413 (281)	
*ERBB2* amplification	Negative	169 (82.8%)	454 (287)	0.46^#^
	Positive	29 (14.2%)	463 (385)	

Number of patients and corresponding median mtDNA content [number of mtDNA molecules per cell] with interquartile range (IQR) for each group and corresponding probabilities (*P* value) for either equal distribution using Mann-Whitney *U* test (#) or Kruskal-Wallis one-way analysis of variance (†). Due to missing values the numbers of samples per variable do not always add up to 204.

### Distant metastasis-free survival and primary tumor mtDNA content

**Figure 1:**
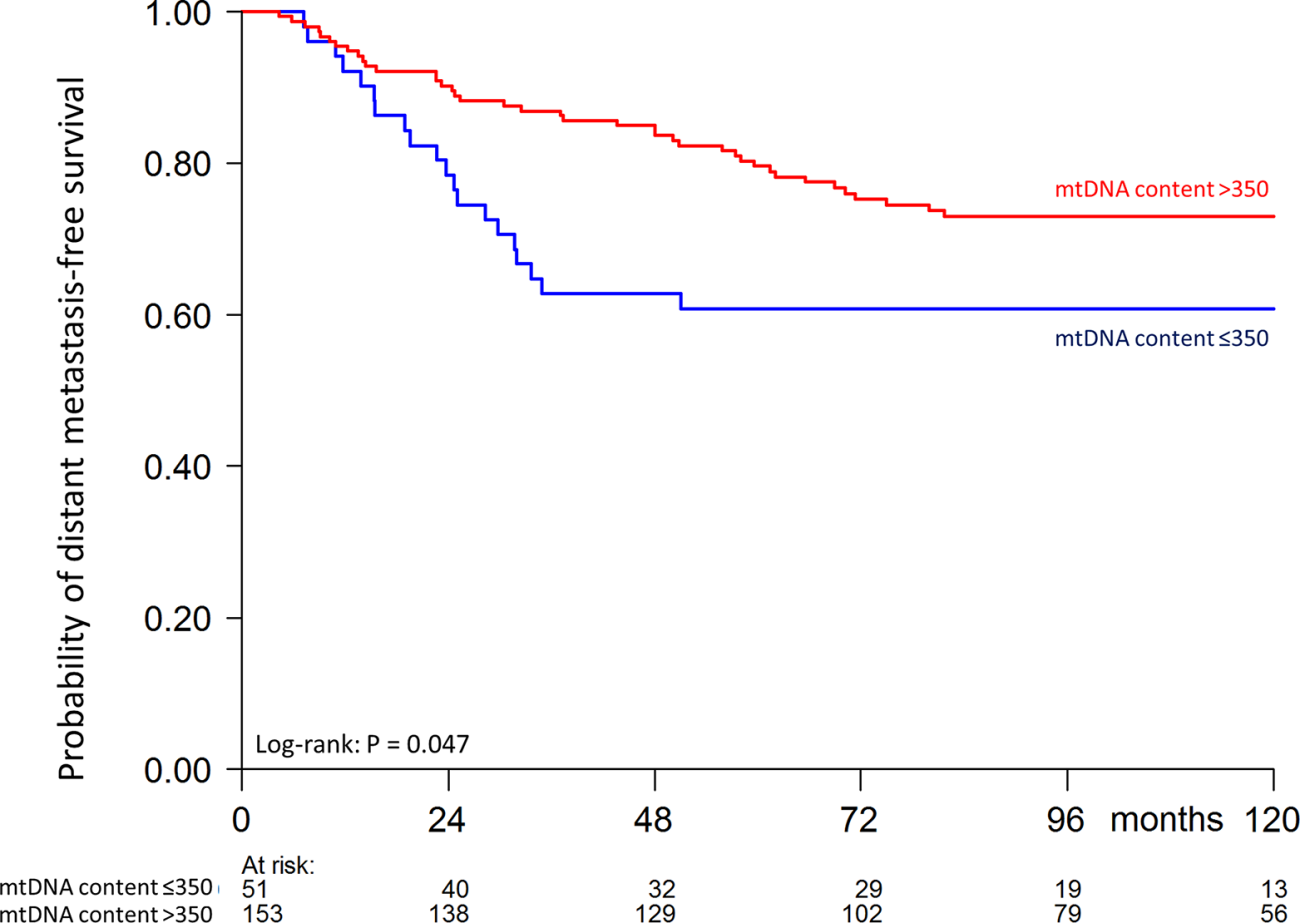
Kaplan-Meier curve showing probability of distant metastasis-free survival as a function of tumor mtDNA content of 204 patients (60 events) Numbers of patients at risk at 24 month time intervals are indicated.

Finally, we studied in our patient cohort the prognostic value of tumor mtDNA content with respect to the length of distant metastasis-free survival. All included breast cancer patients presented as lymph node-negative and did not receive any (neo)adjuvant systemic treatment. The distribution of mtDNA content in our cohort was skewed and could not be normalized by transformation (Skewness and Kurtosis test *P* < 0.05). To assess tumor mtDNA content for the length of metastasis-free survival in our exploratory analysis, we first divided the cohort based on mtDNA content quartiles in four groups (Q1–Q4). Because the patients within the first quarter Q1 presented a different rate of metastasis-free survival compared to the other quarters (Q2–Q4) (Supplementary Figure S1), we divided the cohort in two patient groups of low mtDNA content (Q1 with mtDNA content ≤ 350 mtDNA molecules per cell) versus the rest (Q2–Q4 with mtDNA content > 350 mtDNA molecules per cell). To visualize the length of metastasis-free survival as a function of the levels of tumor mtDNA content (Q1 vs Q2–Q4) we used the Kaplan-Meier survival analysis method (Figure 1). Patients in the low mtDNA content group Q1 showed a higher metastasis probability (log-rank *P* = 0.047). In univariate and multivariable Cox regression analysis including only the 186 patients with no missing values (Table 3), patients in the Q2–Q4 mtDNA content group showed a longer distant metastasis-free survival compared to patients in the low mtDNA tumor content group (univariate: HR 0.50, 95% CI: 0.29–0.87, *P* = 0.015; multivariable: HR 0.54, 95% CI: 0.30–0.97, *P* = 0.038).

**Table 3:** Univariate and multivariable analyses for distant metastasis-free survival in lymph node-negative patients who did not receive any (neo)adjuvant systemic therapy

Variable	Group	*n* (%)	Univariate		Multivariate	
			Hazard ratio (*95% CI*)	*P* value	Hazard ratio (*95% CI*)	*P* value
Age at diagnosis	≤ 40	19 (10.2%)	1		1	
	> 40–55	81 (43.5%)	0.40 (*0.19–0.88*)	0.022	0.34 (*0.15–0.76*)	0.009
	> 55–70	59 (31.7%)	0.42 (*0.19–0.95*)	0.038	0.27 (*0.08–0.92*)	0.037
	> 70	27 (14.5%)	0.39 (*0.14–1.05*)	0.062	0.25 (*0.06–0.97*)	0.045
Menopausal status	Pre	91 (48.9%)	1		1	
	Post	95 (51.1%)	0.94 (*0.55–1.61*)	0.8	1.55 (*0.54–4.44*)	0.4
Tumor size	≤ 2 cm	87 (46.8%)	1		1	
	> 2 cm	99 (53.2%)	1.06 (*0.62–1.83*)	0.8	0.92 (*0.54–1.64*)	0.8
Genomic Grade Index	1	33 (17.7%)	1		1	
	2	56 (30.1%)	1.62 (*0.63–4.18*)	0.3	1.43 (*0.54–3.76*)	0.5
	3	97 (52.2%)	2.65 (*1.03–6.83*)	0.043	2.52 (*0.95–6.66*)	0.063
Progesterone receptor status	Negative	96 (51.6%)	1		1	
	Positive	90 (48.4%)	0.74 (0.38–1.45)	0.4	0.88 (*0.43–1.81*)	0.7
*ERBB2* amplification	Negative	159 (85.5%)	1		1	
	Positive	27 (14.5%)	1.39 (*0.70–2.78*)	0.3	1.45 (*0.70–2.97*)	0.3
mtDNA content	≤ 350	48 (25.8%)	1		1	
	> 350	138 (74.2%)	0.50 (*0.29–0.87*)	0.015	0.54 (*0.30–0.97*)	0.038

Number of patients and corresponding hazard ratio for distant metastasis-free survival with its 95% confidence intervals (CI) and corresponding probabilities for equal risk (*P* value) for each group. Analyses were stratified for estrogen receptor status and limited to the 186 patients (54 events) with no missing values.

## DISCUSSION

Many contradictions about the physiological consequences of reduced mtDNA content exist in the literature. A critical reduction in mtDNA content compromises mitochondrial functioning with downstream effects. Subsequent changes in cellular processes such as aerobic respiration, calcium homeostasis or the intrinsic apoptotic pathway could in turn impact tumorigenic properties. Previous findings have pointed towards a link between low mtDNA content and breast cancer aggressiveness but the exact association remains uncertain. Here, to elucidate its potential as a prognostic marker, the putative relation between tumor mtDNA content and mesenchymal features or distant metastasis-free survival in breast cancer was explored. Using a quantitative PCR approach, the mtDNA content of 41 breast cancer cell lines and 204 primary breast tumor specimens was obtained. A correction for copy number variations of the nuclear-encoded reference locus (HMBS) minimized bias due to tumor-related genomic aberrations in the obtained number of mtDNA molecules per cell. Furthermore, the quantitative mtDNA target in our current assay lies outside of the common deletion region [41] and therefore it is likely that we measure a mixture of functional and dysfunctional mtDNA molecules.

Previous *in vitro* studies reported induction of EMT and stem-cell features upon depletion of mtDNA [12, 14, 19]. In the panel of breast cancer cell lines – homologous cell populations – no relation between mtDNA content and expression levels of genes involved in EMT could be demonstrated. In the cohort of primary breast tumor specimens – more heterogeneous cell populations – we find a positive but weak correlation (ρ ≤ 0.25) between mtDNA content and *ESRP1*, *SNAI1* and *TGFB1*. In *in vitro* studies, a reduction in mtDNA content resulted in decreased ESRP1 protein levels but, contradictory to our findings, increased *SNAI1* mRNA expression or TGFB protein expression. In addition, we could not demonstrate a difference in mtDNA content between the intrinsic subtypes in our cell line panel nor in the cohort of primary breast tumor specimens. Mesenchymal properties have been attributed to the basal and normal-like subtypes, whereas the luminal subtypes are generally epithelial [36, 38, 39]. Accordingly, the evaluated EMT-related genes were commonly highly statistically significant related to each other and differentially expressed between the intrinsic subtypes within our cell line panel and the cohort of primary breast tumor specimens (Supplementary Table S1). Apart from a true lack of association between mtDNA and EMT-features in breast cancer, there are several other reasons which may explain the absence of this association in our data set. To understand the physiological effects of mtDNA content, previous studies suggesting a relation between mtDNA and EMT often used cell lines artificially depleted of mtDNA, termed rho0 clones [42]. The endogenous mtDNA content of the cell lines and primary tumor specimens in our study is a few hundred molecules per cell, which is still orders of magnitude higher than of the rho0 clones. Since the extent of mtDNA reduction is of importance in gaining tumorigenic properties, as demonstrated in glioblastoma models [43], perhaps the mtDNA content in our data set is not at the critically low level necessary to induce a transition towards a mesenchymal phenotype. Alternatively, low mtDNA levels may be important during the process of EMT, but might be restored to normal after the transition is accomplished. Despite the unknown exact reason, we conclude that in our data set mtDNA content is not related to the molecular features connected to a mesenchymal-like phenotype.

To address a possible relation between mtDNA content and aggressive behavior *in vivo*, we analyzed primary breast tumor mtDNA content and prognosis in a cohort of 204 breast cancer patients. Notably, the mtDNA content in our primary tumor specimens is only an estimate, representing not only a heterogeneous tumor cell population but also non-neoplastic cells incorporated in the tumor specimen. However, because no evidence for a relation between mtDNA content and tumor infiltrating lymphocytes [44] was observed (Supplementary Table S2) and stromal content was minimized (Materials and Methods), we estimate the contribution of non-neoplastic cells to the final mtDNA content to be minimal. A few associations between mtDNA content and clinicopathological variables have been reported in previous studies, albeit never consistently [24–29]. These studies included either a low number of study participants or heterogeneous groups regarding treatment regimen or disease stage, making interpretation difficult. In this study, we included a population of lymph node-negative primary breast cancer patients who did not receive any (neo)adjuvant systemic treatment. In this patient group, lower mtDNA content was observed in tumors smaller than 2 cm across compared to tumors larger than 2 cm across. Previous studies could not demonstrate such a difference [27, 28] or reported lower mtDNA content in tumors over 5 cm across compared to smaller tumors [26]. Larger tumors presumably underwent more cell divisions potentially resulting in additional replication-induced mtDNA damage [45], which in turn might require additional compensatory mtDNA molecules to maintain proper mitochondrial functioning. It is also plausible that a hypoxic environment in larger tumors reduces mtDNA content as suggested previously [26]. However, in our primary tumor cohort no relation was observed between mtDNA content and hypoxia-related gene expression [46] as surrogate for the hypoxic state of the tumor (Supplementary Table S2). In addition, our results show a relation between mtDNA content and GGI, a gene expression-based identifier of the histological grade of tumors [40], with the highest grade representing poorly differentiated tumors showing higher mtDNA content. However, we could not demonstrate a conventional significant trend between mtDNA content and GGI. The relation between mtDNA content and histological grade has been reported before [26]. An increase in mtDNA content occurs in early S-phase of the cell cycle [47], and we attribute the relation between grade 3 tumors and higher mtDNA content to the high-proliferative nature of these higher grade tumors. Nevertheless, we note that the median difference in mtDNA content for both tumor size and GGI is only 20%, making a substantial biological consequence of these associations less likely.

Importantly, our cohort is highly suitable to study the prognostic value of mtDNA content for distant metastasis-free survival because all included patients presented with lymph node-negative disease and did not receive any (neo)adjuvant systemic treatment. The size of our cohort did not allow for separate analyzes for estrogen receptor-negative and -positive tumors, which show different proportionality over time (test of proportional hazards assumption *P* = 0.016). Therefore, stratification for estrogen receptor status was applied in all proportional hazard analyses. After adjustment for established prognostic clinicopathological variables, we observed a prognostic effect for mtDNA content. The patients with the 25% lowest mtDNA content (≤ 350 mtDNA molecules per cell) showed a significant unfavorable prognosis with shorter time to metastasis compared to patients with higher mtDNA content. One previous study reported on low tumor mtDNA content corresponding to a higher risk of death [26]. However in that study, no clear information was provided about treatments administered, disease stage at diagnosis and other clinical variables included in their statistical analysis. This renders interpretation and comparison with that previous study difficult. Interestingly, low mtDNA content predicted for a favorable response to anthracycline treatment in a small patient cohort [24]. It is plausible that cells with low mtDNA content are susceptible to such regimen, because damage of mtDNA in cells containing fewer mtDNA molecules can affect mitochondrial functionality more effectively. In our cohort we could study the prognostic value of mtDNA content independent of treatment regimen.

To conclude, we demonstrate a link between particularly low mtDNA content and metastatic potential in breast cancer, which appears to be independent of the mesenchymal phenotype. Low primary tumor mtDNA content potentially identifies patients with unfavorable prognosis but at the same time might predict therapeutic efficacy of DNA-damaging treatment regimen in this group. Larger cohorts of uniformly treated patients are necessary to validate these results and to further unravel the clinical relevance of mtDNA content determination in cancer.

## MATERIALS AND METHODS

### Study cohort and sampling

We employed a panel of 42 breast cancer cell lines (including BT20, BT474, BT483, BT549, CAMA1, DU4475, EVSAT, HCC1937, Hs578T, MCF7, MDAMB134VI, MDAMB157, MDAMB175VII, MDAMB231, MDAMB330, MDAMB361, MDAMB415, MDAMB435s, MDAMB436, MDAMB453, MDAMB468, MPE600, OCUBF, OCUBM, SKBR3, SKBR5, SKBR7, SUM102PT, SUM1315MO2, SUM149PT, SUM159PT, SUM185PE, SUM190PT, SUM225CWN, SUM229PE, SUM44PE, SUM52PE, T47D, UACC812, UACC893, ZR751 and ZR7530 [36, 44]). In addition, DNA extracts from fresh frozen primary breast tumor specimens from an earlier study [37] were selected from our bio-bank at the Erasmus MC. The study was approved by the medical ethics committee of the Erasmus MC (MEC 02.953) and conducted in accordance to the code of conduct of Federation of Medical Scientific Societies in the Netherlands. Whenever possible, we adhered to the Reporting Recommendations for Tumor Marker Prognostic Studies (REMARK) [48]. Patient selection criteria have been described before [49] and include lymph node-negative primary breast cancer with local treatment but no systemic (neo)adjuvant therapies. Our selection (Supplementary Figure S2) was based on availability of genotypic data and gene expression data from the primary tumors (*n* = 337) and availability of uniformly extracted DNA (see below) (*n* = 250). Next, specimens with a tumor cell percentage below 50% were excluded to minimize skewed values due to stromal cell contamination (*n* = 38). In addition, five patient samples were ineligible in retrospect and excluded. Thus, mtDNA content was examined in a total of 207 patients. Patients' follow-up involved examinations every 3 months for the first two 2 years, every 6 months for years 3–5, and every 12 months from year 5 onwards. Estrogen receptor and progesterone receptor status were determined as described before [50]. Evaluation of *ERBB2* amplification via RNA expression levels and the qRT-PCR Genomic Grade Index were determined as described before respectively [51] and [40].

### DNA extraction

DNA was extracted from cultured cell lines using the DNeasy Blood & Tissue kit (*Qiagen, Venlo, Netherlands*) according the suppliers' protocol. We selected the DNA which was previously extracted from cryostat sections of the primary tumor tissues based on uniformity in extraction procedure (*QIAamp* DNA mini kit (*Qiagen*) as described before [37]). DNA extracts were quantified using the Qubit dsDNA HS assay kit (*Life Technologies, Carlsbad, United States of America*) and all samples were diluted to a concentration of 0.2 ng/μL DNA prior to mtDNA content analysis.

### Copy number analysis

Copy number variation of the nuclear encoded *HMBS* gene – which served as a reference to obtain mtDNA content – was obtained from our previously described microarray data (Gene Expression Omnibus database accession numbers GSE10099 [37] and GSE41308 [52]). The breast cancer cell lines were genotyped on the Genome Wide Human SNP Array 6.0 (*Affymetrix, Santa Clara, United States of America*), the primary tumor specimens on the GeneChip Human Mapping 100K SNP Array (*Affymetrix*).

### mtDNA content

Mitochondrial DNA content was determined in duplicate runs using a multiplex quantitative PCR targeting the nuclear *HMBS* gene (chr11q23.2-qter) and the mitochondrial *MT-TL1* (chrMT 3212–3319). Primers targeting the nuclear encoded *HMBS* gene (forward 5′-TGAGGCGGATGCAGATAC-3′ and reverse 5′- CCCACCCACGGTAGTAATTC-3′ (*Life technologies*)) yielded a 201 bp amplicon quantitatively detected using a CY5 labeled probe (5′-[CY5]TATCAGCCAAGCCTCCGAAC[BHQ2]-3′ (*Sigma Aldrich, St. Louis, United States of America*)). Primers targeting the mitochondrial encoded *MT-TL1* (forward 5′-CACCCAAGAACAGGGTTTGT-3′ and reverse 5′-TGGCCATGGGTATGTTGTTA-3′ (*Life Technologies*)) yielded a 108 bp amplicon quantitatively detected using a HEX labeled probe (5′-[HEX]TTACCGGGCTCTGCCATCT[BHQ1]-3′, (*Sigma Aldrich*)) [53]. Reactions included 1x Absolute QPCR Mix containing SYBR Green and ROX (AB-1163 *Life Technologies*) in the presence of 100 nM mtDNA primers, 360 nM nDNA primers and 100 nM probes. The 45-cycle PCR was carried out at a 62°C annealing temperature and probe fluorescence was monitored using ROX, HEX, CY5 and FAM filters on Mx3000P or Mx3005P qPCR systems (*Agilent Technologies, Waldbronn, Germany*). Quantification cycle values (Cq [dRN]) were obtained using the adaptive baseline approach (MxPro v4.10) up to cycle 35 with fixed fluorescence thresholds at 0.004 dRn. Performance of singleplex PCR and multiplex PCR runs was comparable (Supplementary Table S3). Performance of the assay at variable ratios of artificial *HMBS* (289 bp linear: ACA GAC GGG GTC CTT TCA TTC GAG GCT GGG CTG AGG CGG ATG CAG ATA CGG CCC CTT TGG GAA GAC ACG TTC CAC TTT TGA TTC ATA GGA GAG AGT ATC AGC CAA GCC TCC GAA CTG CAC ACA AAC GTC TTA GAA GTG CGC CTT CTT TTT GTG TTA TAG TGG TCT CCC AGC CAC AGC CAA CGC TCC AAG TCC CCA GCT GTG ACA CAC CTA CTG AAT TAC TAC CGT GGG TGG GAG GCC GCC GTG GGC CTT TCC ATT ACG AGC CTG CTT GCC GAG CCC TGG GCT TGT GCA C) and artificial MT-TL1 (180 bp cloned in circular 2374 bp pMA-T vector: TAT CAT CTC AAC TTA GTA TTA TAC CCA CAC CCA CCC AAG AAC AGG GTT TGT TAA GAT GGC AGA GCC CGG TAA TCG CAT AAA ACT TAA AAC TTT ACA GTC AGA GGT TCA ATT CCT CTT CTT AAC AAC ATA CCC ATG GCC AAC CTC CTA CTC CTC ATT GTA CCC ATT CTA ATC GCA ATG GCA) was linear (Supplementary Table S4). DNA input of the breast cancer cell lines and the primary breast tumor specimens was standardized for 1 ng DNA per reaction. A calibration curve containing a pool of DNA isolates from independent fresh frozen tumors was taken along as internal control to monitor inter assay variation. Obtained Cq values were used to calculate the ratio of mitochondrial DNA opposed to nuclear DNA by the relative quantitation method (2^ΔCq [54]). Multiplying this ratio by the copy number of *HMBS* (obtained as described above) resulted in the number of mtDNA molecules per cell as mtDNA content.

### Gene expression analysis

Gene expression data of the cell lines was obtained from our previously described triplicate microarray data (Gene Expression Omnibus database accession number GSE41313 [52]) on the Human Genome HT_HG-U133_Plus_PM GeneChip 96-well arrays (*Affymetrix*). Data of all breast cancer cell lines were available with the exception of SUM225CWN. Gene expression data of the primary breast tumor specimens was obtained from our previously described microarray data (Gene Expression Omnibus database accession number GSE2034 [50] and GSE5327 [55]) on the Human Genome HG-U133a GeneChip 96-well arrays (Affymetrix). Subtype classification was based on expression of the intrinsic gene set defined by Perou et al. [56]. Cell line DU4475 could not be classified to a subtype group and was therefore excluded from the intrinsic subtype analysis. For individual genes, levels based on log2 transformed distances to the geometric mean for each probe set were obtained for probe IDs 201131_s_at (*CDH1*), 203440_at (*CDH2*), 219121_s_at (*ESRP1*), 210495_x_at (*FN1*), 203936_s_at (*MMP9*), 219480_at (*SNAI1*), 213139_at (*SNAI2*), 203085_s_at (*TGFB1*), 213943_at (*TWIST1*) and 201426_s_at (*VIM*). The tumor infiltrating lymphocyte classification as low TIL and high TIL was based on the immune signature probe-set by Massink et al. [44]. Classification as low hypoxia response and high hypoxia response was based on the expression of the hypoxia-related gene signature as described before by Chi et al. [57].

### Statistical analyses

All analyses included the average mtDNA content obtained from the duplicate analysis for each individual sample. Data distribution was tested using Skewness-Kurtosis tests for normality. Numerical correlations between RNA expression levels and mtDNA content were investigated using the Spearman rank correlation and corrected for multiple testing using the false discovery rate controlling procedure [58]. Categorical comparisons of the intrinsic subtypes or grouped clinical variables and mtDNA content were employed using either Mann-Whitney *U*-tests (two groups) or Kruskal-Wallis one-way analysis of variance (multiple groups). When appropriate, we performed Cuzick's test for trend across ordered categorical variables. Kaplan-Meier survival plots and log-rank tests were used to assess the differences in time to distant metastasis between mtDNA content groups. Proportional hazard analyses for distant metastasis-free survival were performed using Cox proportional-hazards regression methods. We stratified for estrogen receptor status and censored for 10 years clinical follow-up (most patients are redirected to their general practitioner at that point in time) to maintain proportionality (test of proportional hazards assumption using the Schoenfeld residuals *P* > 0.05). Univariate analysis was done on the individual clinicopathological variables, multivariable analysis included all clinicopathological variables and mtDNA content. All statistical tests were two-sided, and *P* values smaller than 0.05 were considered as statistically significant. Clinical variables were statistically analyzed in Stata version 13.1 (*StataCorp LP, College Station, United States of America*). Other analyses were performed using Spotfire 7.0.0 (*TIBCO, Palo Alto, United States of America*).

## SUPPLEMENTARY MATERIALS FIGURES AND TABLES


