# Supplementary material for: Mitochondrial DNA content in breast cancer: Impact on in vitro and in vivo phenotype and patient prognosis
Source: Oncotarget. 2016 Apr 11;7(20):29166–76. doi: 10.18632/oncotarget.8688 (PMC5045386; doi:10.18632/oncotarget.8688)
Supplement: Supplementary file 1 [file oncotarget-07-29166-s001.pdf]

# Mitochondrial DNA content in breast cancer: Impact on *in vitro* and *in vivo* phenotype and patient prognosis

## Supplementary Materials

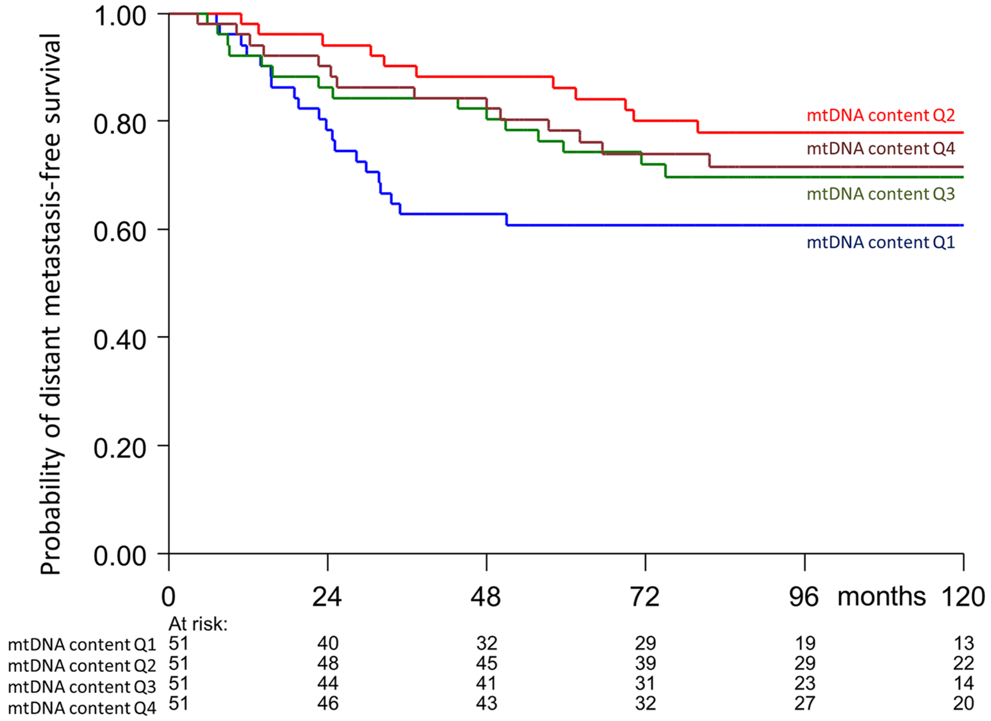


**Supplementary Figure S1: Kaplan-Meier curve showing probability of distant metastasis-free survival as a function of tumor mtDNA content of 204 patients (60 events).** Numbers of patients at risk at 24 month time intervals are indicated.

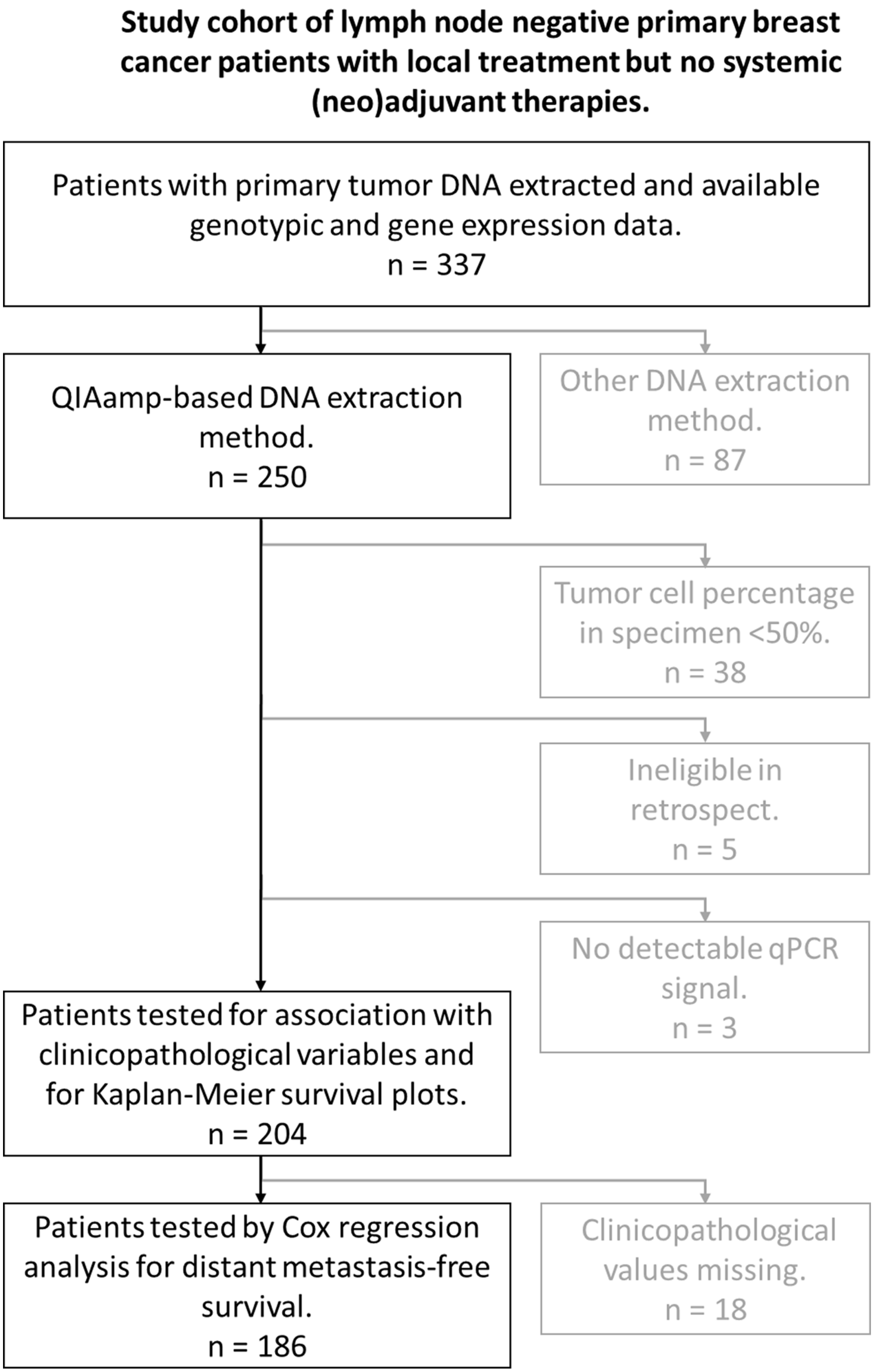


**Supplementary Figure S2: REMARK diagram for mtDNA content study cohort.**

**Supplementary Table S1A: Relation between mtDNA content and the mesenchymal phenotype in breast cancer cell lines**

| | | **CDH1** | **CDH2** | **ESRP1** | **FN1** | **MMP9** | **SNAI1** | **SNAI2** | **TGFB1** | **TWIST1** | **VIM** | **mtDNA content** |
|---|---|---|---|---|---|---|---|---|---|---|---|---|
| $\rho$ | CDH1† | | | | | | | | | | | *0.07* |
| *P* value | | | | | | | | | | | | 6.6E–01 |
| $\rho$ | CDH2† | *−0.38* | | | | | | | | | | *−0.01* |
| *P* value | | **1.6E–02** | | | | | | | | | | 9.7E–01 |
| $\rho$ | ESRP1† | *0.63* | *−0.32* | | | | | | | | | *0.04* |
| *P* value | | **1.0E–05** | 4.4E–02 | | | | | | | | | 8.0E–01 |
| $\rho$ | FN1† | *−0.42* | *0.44* | *−0.47* | | | | | | | | *−0.04* |
| *P* value | | **6.4E–03** | **4.9E–03** | 2.2E–03 | | | | | | | | 8.0E–01 |
| $\rho$ | MMP9† | *0.43* | *−0.47* | *0.30* | *0.09* | | | | | | | *0.13* |
| *P* value | | **6.1E–03** | **2.3E–03** | 6.2E–02 | 6.0E–01 | | | | | | | 4.4E–01 |
| $\rho$ | SNAI1† | *−0.16* | *0.02* | *−0.26* | *0.17* | *−0.29* | | | | | | *−0.09* |
| *P* value | | 3.3E-01 | 8.8E–01 | 9.9E–02 | 3.1E–01 | 6.6E–02 | | | | | | 6.0E–01 |
| $\rho$ | SNAI2† | *−0.34* | *0.61* | *−0.37* | *0.39* | *−0.32* | *−0.09* | | | | | *−0.08* |
| *P* value | | 3.1E–02 | **3.1E–05** | **2.0E–02** | **1.3E–02** | 4.6E–02 | 5.6E–01 | | | | | 6.4E–01 |
| $\rho$ | TGFB1† | *−0.44* | *0.48* | *−0.60* | *0.58* | *−0.31* | *0.23* | *0.58* | | | | *−0.30* |
| *P* value | | **4.9E–03** | **1.9E–03** | **5.0E–05** | **8.0E–05** | 5.5E–02 | 1.6E–01 | **8.2E–05** | | | | 5.7E–02 |
| $\rho$ | TWIST1† | *−0.36* | *0.60* | *−0.26* | *0.40* | *−0.28* | *0.15* | *0.32* | *0.42* | | | *−0.18* |
| *P* value | | **2.3E–02** | **3.8E–05** | 1.1E–01 | **1.0E–02** | 8.4E–02 | 3.7E–01 | 4.3E–02 | **6.4E–03** | | | 2.7E–01 |
| $\rho$ | VIM† | *−0.54* | *0.60* | *−0.57* | *0.54* | *−0.50* | *0.17* | *0.44* | *0.61* | *0.56* | | *−0.31* |
| *P* value | | **3.7E–04** | **5.1E–05** | **1.3E–04** | **3.0E–04** | **1.1E–03** | 3.0E–01 | **4.3E–03** | **3.2E–05** | **1.5E–04** | | 5.3E–02 |
| *P* value | Subtype‡ | **2.6E–04** | **2.3E–04** | **5.6E–05** | **7.5E–03** | **1.6E–02** | 6.4E–01 | **1.6E–04** | **1.6E–05** | **2.2E–03** | **7.4E–06** | 1.4E–01 |

Data are correlation coefficients ($\rho$) and corresponding probabilities for independence (*P* value) using Spearman's Rank correlation (†) or probabilities (*P* value) for equal distribution using Kruskal-Wallis one-way analysis of variance (‡) between RNA expression levels (indicated genes), intrinsic subtypes, or mtDNA content of the breast cancer cell lines. *P* values in bold indicate statistical significance after FDR correction of 5% ($P < 0.027$).

## Supplementary Table S1B: Relation between mtDNA content and the mesenchymal phenotype in primary breast tumor specimens

| | | CDH1 | CDH2 | ESRP1 | FN1 | MMP9 | SNAI1 | SNAI2 | TGFB1 | TWIST1 | VIM | mtDNA content |
|---|---|---|---|---|---|---|---|---|---|---|---|---|
| *ρ* | CDH1† | | | | | | | | | | | *0.05* |
| *P* value | | | | | | | | | | | | **4.4E–01** |
| *ρ* | CDH2† | *−0.10* | | | | | | | | | | *−0.04* |
| *P* value | | 1.7E–01 | | | | | | | | | | 5.3E–01 |
| *ρ* | ESRP1† | 0.03 | −0.11 | | | | | | | | | *0.25* |
| *P* value | | 6.7E–01 | 1.1E–01 | | | | | | | | | **2.4E–04** |
| *ρ* | FN1† | *−0.07* | *0.36* | *−0.18* | | | | | | | | *0.04* |
| *P* value | | 3.3E–01 | **1.6E–07** | **1.0E–02** | | | | | | | | 5.2E–01 |
| *ρ* | MMP9† | *−0.10* | *0.21* | *0.07* | *0.16* | | | | | | | *0.00* |
| *P* value | | 1.6E–01 | **2.3E–03** | 3.2E–01 | **2.5E–02** | | | | | | | 9.8E–01 |
| *ρ* | SNAI1† | *−0.11* | *0.14* | *0.24* | *0.10* | *0.13* | | | | | | *0.23* |
| *P* value | | 1.3E–01 | 4.6E–02 | **6.6E–04** | 1.7E–01 | 7.2E–02 | | | | | | **9.4E–04** |
| *ρ* | SNAI2† | *−0.25* | *0.09* | *−0.18* | *0.36* | *0.26* | *0.04* | | | | | *−0.05* |
| *P* value | | **3.5E–04** | 2.1E–01 | **1.2E–02** | **8.6E–08** | **1.7E–04** | 5.3E–01 | | | | | 4.4E–01 |
| *ρ* | TGFB1† | *−0.05* | *−0.06* | *0.13* | *0.20* | *0.23* | *0.14* | *0.26* | | | | *0.18* |
| *P* value | | 5.2E–01 | 3.6E-01 | 6.5E–02 | **3.7E–03** | **7.3E–04** | 4.2E–02 | **2.1E–04** | | | | **8.5E–03** |
| *ρ* | TWIST1† | *−0.21* | *0.25* | *−0.32* | *0.41* | *0.18* | *0.00* | *0.43* | *−0.02* | | | *−0.15* |
| *P* value | | **2.1E–03** | **2.4E–04** | **2.8E–06** | **9.0E–10** | **1.2E–02** | 9.8E–01 | **8.8E–11** | 7.2E–01 | | | 2.7E–02 |
| *ρ* | VIM† | *−0.40* | *0.26* | *−0.11* | *0.35* | *0.28* | *0.22* | *0.57* | *0.30* | *0.31* | | *−0.05* |
| *P* value | | **2.7E–09** | **1.3E–04** | 1.3E–01 | **3.0E–07** | **4.1E–05** | **1.3E–03** | **9.5E–19** | **1.6E–05** | **5.5E–06** | | 4.7E–01 |
| *P* value | Subtype‡ | **6.8E–07** | **2.6E–02** | **3.9E–06** | **5.9E–03** | **1.5E–05** | **3.5E–05** | **6.9E–06** | **1.6E–02** | 2.8E–02 | **9.4E–09** | 8.2E–01 |

Data are correlation coefficients (*ρ*) and corresponding probabilities for independence (*P* value) using Spearman's Rank correlation (†) or probabilities (*P* value) for equal distribution using Kruskal-Wallis one-way analysis of variance (‡) between RNA expression levels (indicated genes), intrinsic subtypes, or mtDNA content of the primary breast tumor specimens. *P* values in bold indicate statistical significance after FDR correction of 5% ($P < 0.027$).

**Supplementary Table S2: Gene expression signatures in relation to mtDNA content in primary breast tumor specimens**

| Gene signature | Group | *n*(%) | mtDNA content (IQR) | *P*-value |
|---|---|---|---|---|
| Tumor infiltrating lymphocytes [44] | Low TIL | 140 (68.6%) | 502 (348) | 0.08# |
| | High TIL | 64 (31.4%) | 424 (282) | |
| Hypoxia [46] | Low hypoxia response | 96 (47.1%) | 475 (251) | 0.6# |
| | High hypoxia response | 108 (52.9%) | 458 (343) | |

Number of patients and corresponding median mtDNA content [number of mtDNA molecules per cell] with interquartile range (IQR) for each group and corresponding probabilities (*P* value) for equal distribution using Mann-Whitney *U* test (#).

**Supplementary Table S3: Performance of qPCR assay targeting *HMBS* and *MT-TL1* in singleplex and multiplex format**

| | | | Singleplex | Multiplex |
|---|---|---|---|---|
| mtDNA signal | DNA [ng/well] | 0.0038 | 32.50 (0.01) | 30.31 (0.03) |
| nDNA signal | | | - | - |
| mtDNA signal | | 0.038 | 27.12 (0.05) | 27.01 (0.00) |
| nDNA signal | | | - | - |
| mtDNA signal | | 0.38 | 23.29 (0.23) | 23.47 (0.02) |
| nDNA signal | | | 33.20 (0.09) | 33.23 (0.11) |
| mtDNA signal | | 3.8 | 19.90 (0.06) | 20.02 (0.02) |
| nDNA signal | | | 29.84 (0.06) | 29.73 (0.02) |
| mtDNA signal | | 38 | 16.71 (0.15) | 16.61 (0.01) |
| nDNA signal | | | 26.17 (0.13) | 26.32 (0.27) |
| mtDNA signal | | 380 | 14.16 (0.28) | 14.25 (0.01) |
| nDNA signal | | | 23.04 (0.00) | 23.09 (0.06) |
| mtDNA | ***Efficiency ($R^2$)*** | | *89.3% (0.984)* | *101.6% (0.997)* |
| nDNA | | | *96.3% (0.999)* | *97.5% (0.999)* |

Calibration curves containing 0.0038–380 ng DNA per reaction were run in duplicate singleplex or multiplex assays, the obtained quantification cycle values [mean Cq (standard deviation)] for the qPCR assays targeting mtDNA and nDNA are indicated per reaction. The performance is evaluated by corresponding efficiency and $R^2$ of the data points.

**Supplementary Table S4: Performance of qPCR assay targeting artificial *HMB*S and *MT-TL1* at variable ratio's**

| | | | Artificial *HMBS* [ng/well] | |
|---|---|---|---|---|
| | | | **0.002** | **0.02** |
| mtDNA signal | Artificial *MT-TL1* [ng/well] | 0.006 | 26.84 (0.36) | ND |
| nDNA signal | | | 25.53 (0.37) | |
| mtDNA signal | | 0.063 | ND | 23.07 (0.07) |
| nDNA signal | | | | 22.13 (0.16) |
| mtDNA signal | | 0.63 | 21.63 (0.07) | ND |
| nDNA signal | | | 25.38 (0.11) | |
| mtDNA signal | | 0.64 | 20.59 (0.09) | ND |
| nDNA signal | | | 25.27 (0.03) | |
| mtDNA signal | | 3.20 | 18.05 (0.02) | 18.15 (0.02) |
| nDNA signal | | | 25.40 (0.11) | 21.91 (0.16) |
| mtDNA signal | | 6.29 | 17.32 (0.13) | 17.16 (0.24) |
| nDNA signal | | | 25.65 (0.17) | 21.70 (0.06) |
| mtDNA signal | | 6.40 | 16.73 (0.11) | ND |
| nDNA signal | | | 25.69 (0.12) | |
| mtDNA signal | | 32.00 | 15.23 (0.06) | 14.64 (0.30) |
| nDNA signal | | | 25.53 (0.06) | 22.41 (0.19) |
| mtDNA signal | | 64.00 | 13.36 (0.32) | 13.36 (0.04) |
| nDNA signal | | | 25.99 (0.01) | 22.30 (0.32) |
| mtDNA signal | | 62.86 | ND | 14.37 (0.15) |
| nDNA signal | | | | 22.37 (0.01) |
| mtDNA signal | | 320.00 | 12.17 (0.18) | 12.46 (0.13) |
| nDNA signal | | | 25.80 (0.11) | 22.55 (0.21) |
| mtDNA signal | | 640.00 | ND | 11.79 (0.40) |
| nDNA signal | | | | 22.31 (0.23) |
| mtDNA | *Efficiency ($R^2$)* | | *111.2% (0.988)* | |
| nDNA | | | *98.0% (0.975)* | |

Variable input of artificial *HMBS* and *MT-TL1* constructs were run in duplicate multiplex assays, the obtained quantification cycle values [mean Cq (standard deviation)] for the qPCR assays targeting mtDNA and nDNA are indicated per reaction. The performance is evaluated by corresponding efficiency and $R^2$ of the data points.
